# Robust antiviral activity of commonly prescribed antidepressants against emerging coronaviruses: in vitro and in silico drug repurposing studies

**DOI:** 10.1038/s41598-022-17082-6

**Published:** 2022-07-28

**Authors:** Omnia Kutkat, Yassmin Moatasim, Ahmed A. Al‐Karmalawy, Hamada S. Abulkhair, Mokhtar R. Gomaa, Ahmed N. El-Taweel, Noura M. Abo Shama, Mohamed GabAllah, Dina B. Mahmoud, Ghazi Kayali, Mohamed A. Ali, Ahmed Kandeil, Ahmed Mostafa

**Affiliations:** 1 Center of Scientific Excellence for Influenza Viruses, National Research Centre, Dokki, Giza, 12622 Egypt; 2 Department of Pharmaceutical Chemistry, Faculty of Pharmacy, Horus University-Egypt, New Damietta, 34518 Egypt; 3 Department of Pharmaceutical Organic Chemistry, Faculty of Pharmacy, Al-Azhar University, Nasr City, Cairo, 11884 Egypt; 4 Pharmaceutics Department, Egyptian Drug Authority, formerly known as National Organization for Drug Control and Research, Giza, 12654 Egypt; 5 Department of Epidemiology, Human Genetics, and Environmental Sciences, University of Texas, Houston, TX 77030 USA; 6 Human Link DMCC, Dubai, UAE

**Keywords:** Drug discovery, Diseases, Chemistry

## Abstract

During the current coronavirus disease 2019 (COVID-19) pandemic, symptoms of depression are commonly documented among both symptomatic and asymptomatic quarantined COVID-19 patients. Despite that many of the FDA-approved drugs have been showed anti-SARS-CoV-2 activity in vitro and remarkable efficacy against COVID-19 in clinical trials, no pharmaceutical products have yet been declared to be fully effective for treating COVID-19. Antidepressants comprise five major drug classes for the treatment of depression, neuralgia, migraine prophylaxis, and eating disorders which are frequently reported symptoms in COVID-19 patients. Herein, the efficacy of eight frequently prescribed FDA-approved antidepressants on the inhibition of both SARS-CoV-2 and MERS-CoV was assessed. Additionally, the in vitro anti-SARS-CoV-2 and anti-MERS-CoV activities were evaluated. Furthermore, molecular docking studies have been performed for these drugs against the spike (S) and main protease (M^pro^) pockets of both SARS-CoV-2 and MERS-CoV. Results showed that Amitriptyline, Imipramine, Paroxetine, and Sertraline had potential anti-viral activities. Our findings suggested that the aforementioned drugs deserve more in vitro and in vivo studies targeting COVID-19 especially for those patients suffering from depression.

## Introduction

Coronavirus (CoV) is composed of an enveloped capsid with positive sense non-segmented single-stranded RNA (+ ssRNA) genome (26–32 kb) in the core. They are categorized into four main genera, designated as alpha-, beta-, gamma-, and delta-CoVs. Taxonomically, CoVs reside in subfamily Coronavirinae, family Coronaviridae and order Nidovirales^1^. For a long time, two alpha-CoVs (HCoV-229E and HCoV-NL63) and two beta-CoVs (HCoV-OC43 and HCoV-HKU1) could solely escape the species barrier, causing a mild and self-limiting respiratory infection in humans which is commonly known as “common cold”. This small collection of human-infecting coronaviruses was further extended to include Severe Acute Respiratory Syndrome Coronavirus (SARS-CoV) and the Middle East Respiratory Syndrome Coronavirus (MERS-CoV) in 2003, and 2012, respectively^1^. These two viruses are attributed to highly pathogenic human beta-CoVs^1^.

In late December 2019, the WHO Office in China was informed of pneumonia cases of unidentified etiology in Wuhan City which were subsequently declared to be associated with a new coronavirus, later known as Severe Acute Respiratory Syndrome Coronavirus 2 (SARS-CoV-2). The devastating expansion of SARS-CoV-2 was faster than any other zoonotic coronavirus^2^. This urged the need to find medications to reduce mortality and the unaffordable hospitalization rates, especially in the absence of effective prepandemic vaccines or antiviral therapeutics. Several trials were performed to re-purpose FDA-approved drugs as a faster way to discovering effective drugs in COVID-19 treatment protocol^3–8^. In addition, computational methods are very common in drug design and discovery processes. Molecular docking and dynamics simulations are the most widely used processes that help in the discovery of new drug candidates or the introduction of new drug uses for already approved drugs through repurposing processes^9–11^.

Several studies reported that a considerable percentage of COVID-19 patients and healthcare workers were prone to develop psychological health issues including infection-related and lockdown-related depressions, anxiety, or dementia^12–15^. FDA-approved antidepressants are prescribed in certain psychological disorders, including major depressive disorder, anxiety, bipolar disorder and less commonly in attention deficit hyperactivity disorder (ADHD)^16^. Moreover, antidepressants could also be prescribed to prevent migraines, treat neuropathic pain, and less commonly insomnia.

Antidepressants are categorized into five classes including the tricyclic antidepressants (TCA), the selective serotonin re-uptake inhibitors (SSRIs), the selective serotonin and norepinephrine re-uptake inhibitors (SNRIs), the monoamine oxidase inhibitors (MAOIs), and atypical antidepressants that include unclassified drugs (e.g. bupropion, Mirtazapine and vortioxetine). Some antidepressants were experimentally tested to be repurposed for the management of bacterial and fungal infections^17^. Recently, the antiviral activity of imipramine hydrochloride, sertraline hydrochloride, amitriptyline hydrochloride, and paroxetine hydrochloride have been tested against Marburg Virus^18^ and paroxetine against SARS-CoV-2^19^.

**Figure 1:**
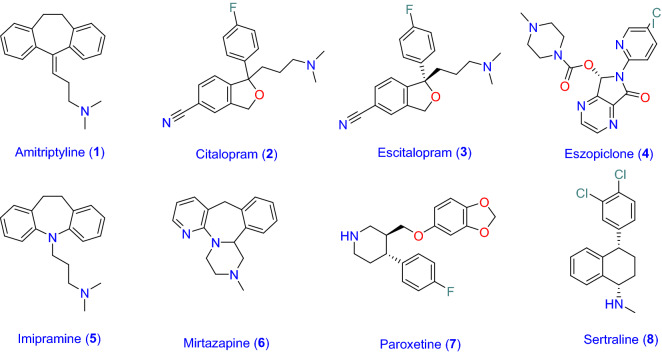
FDA-approved antidepressants with potential antiviral activity.

Encouraged by the above pieces of evidence and in line with the global impetus of finding a new effective antiviral agent to treat the pandemic SARS-CoV-2, we assessed the anti-SARS-CoV-2 activity of eight commonly prescribed antidepressants (1–8) including Amitriptyline and Imipramine (TCAs class), Citalopram, Escitalopram, Paroxetine, Sertraline, Mirtazapine (SSRIs class), and Eszopiclone (MAOIs class) (Fig. 1). Furthermore, the antiviral activities of promising anti-SARS-CoV-2 antidepressant drugs were evaluated against MERS-CoV to determine their antiviral spectrum.

## Materials and methods

### Viruses, cells, and tested drugs

Vero-E6 cells were cultured in Dulbecco's Modified Eagle's medium (DMEM), supplemented with 10% Fetal Bovine Serum (FBS) (Invitrogen) and 1% Penicillin/Streptomycin mixture (pen/strep) and incubated at 37 °C in a humidified incubator and 5% CO_2_.

The hCoV-19/Egypt/NRC-3/2020 SARS-CoV-2 “NRC-03-nhCoV” virus^20^ and the MERS-CoV isolate NRCE-HKU270^21^ were propagated in Vero-E6 cells, as described previously with minor modifications^8,22^. Briefly, confluent VERO-E6 cell monolayers were infected with NRC-03-nhCoV or NRCE-HKU270 in infection medium (DMEM with 1% pen/strep, 0.2% Bovine Serum Albumin (BSA), and 1 µg/ml TPCK-treated trypsin) at a multiplicity of infection (MOI) of 0.1 for 2 h. Following virus adsorption, the infection medium containing virus inoculum was discarded and exchanged with new infection media. The infected VERO-E6 monolayers were incubated for 4 days post-infection at 37 °C in a humidified incubator and 5% CO_2_. The cell supernatants were harvested and centrifuged at 2500 rpm for at least 5 min to remove cellular remains. The clear supernatant containing propagated viruses were then aliquoted, preserved at − 80 °C freezer. An aliquot of the saved NRC-03-nhCoV or NRCE-HKU270 viruses were subjected to virus titration using plaque titration assay, as described previously^8,23^.

The tested antidepressant FDA-approved drugs; Citalopram, Escitalopram, Paroxetine, Sertraline, Mirtazapine, Amitriptyline, Imipramine, and Eszopicolone; were kindly provided by the Egyptian Holding Company for Pharmaceuticals and Chemicals, and the Egyptian National Organization for Drug Control and Research in Egypt.

### In vitro antiviral bioassay

#### Half maximal cytotoxic concentration (CC_50_) determination

To assess CC_50_ of the selected antidepressants, stock solutions were prepared by dissolving the selected drugs in 10% DMSO “in 1X DMEM” and serially diluted them with 1X DMEM to prepare the various working concentrations (Concentration range = 1–1000 µMol). The CC_50_ of each compound was assayed in Vero-E6 cells by using crystal violet assay as previously described^24^. Briefly, 100 µl of the VERO-E6 cell suspension were distributed into 96-well plates (3*10^5^ cells/mL). The seeded plates were then incubated at 37 °C in 5% humidified CO_2_ incubator for 24 h. Cell monolayers were then co-incubated with different concentrations of each drug in triplicates at 37 °C in 5% humidified CO_2_ incubator. Seventy two hours later, the media supernatants were discarded, the cell monolayers were washed once with 1X PBS and fixed with 10% formaldehyde for 1 h at room temperature (RT). The plates were further dried and stained at RT with 0.1% crystal violet for 20 min on a bench rocker. The monolayers are then washed, dried, and the crystal violet dye in each well was then dissolved with 200 µL methanol for 20 min on a bench rocker at RT. Eventually, the absorbance was measured at λmax 570 nm using the Anthos Zenyth 200rt plate reader (Anthos Labtec Instruments, Heerhugowaard, Netherlands). The cytotoxicity of various concentrations compared to the untreated cells was determined using nonlinear regression analysis by plotting log inhibitor versus normalized response.

#### Inhibitory concentration 50 (IC_50_)

The IC_50_ values for the tested compound were determined as previously described^8^, with minor modifications. Briefly, the VERO-E6 monolayers in 96-well tissue culture plates were then washed once with 1 × PBS. The NRC-03-nhCoV” virus “TCID_50_ = 100) was co-incubated with serial diluted working concentrations of the tested drugs at 37 °C for 1 h. The Vero-E6 cells were treated with virus/drug mixtures and kept at 37 °C for 1 h. Untreated/infected cells represented the virus control, however untreated/uninfected cells referred to the cell control. After 72 h of co-incubation at 37 °C in 5% CO_2_ incubator, the cell monolayers were fixed with 100 μL of 10% formaldehyde for 20 min and stained with 0.1% crystal violet “in distilled water” for 15 min at RT. To dissolve crystal violet dye, 100 μL of the absolute methanol were added per well and the optical density of the color is eventually measured at 570 nm using the Anthos Zenyth 200rt plate reader (Anthos Labtec Instruments, Heerhugowaard, Netherlands). The IC_50_ values were calculated using nonlinear regression analysis by plotting log inhibitor versus normalized response.

#### Quantitative real time RT-PCR assessment mRNA expression

Vero-E6 cell suspensions were cultured in 12-well tissue culture plates and incubated overnight to get confluent monolayers. The cells were washed two times with 1 × PBS and 100 µL of virus HCoV-19/Egypt/NRC-1/2020 with dilution of MOI = 0.05 was preincubated for 1 h at 37 °C with 5% CO_2_ before being added to the cells with 100 µL of predefined non-cytotoxic concentrations of the selected compounds. The compounds/virus mixtures were then added to the corresponding wells. Treated wells, cell control, and virus control are incubated at 37 °C under 5% CO_2_ for 1 h with rocked every l5 min to ensure homogenous exposure of the cells to infection and avoid drying of cells. After 1 h, inoculum was removed and followed by addition of 1000 µL of infection medium, then infected cells were incubated at 37 °C with 5% CO_2_ for 48 h. An aliquot of 300 µL was collected every 24 h from each dilution in duplicate for viral titration using qRT-PCR.

A part of the sample underwent viral RNA extraction using the QIAamp Viral-RNA Kit (Qiagen, Germany). The qRT-PCR assay (targeting ORF1b-nsp14 gene) in the presence of specific primers and probes were performed using Verso 1-step qRT-PCR Kit (Thermo, USA). The primer and probe sequences for the ORF1b gene assay are: 5′-TGGGGYTTTACRGGTAACCT-3′ (Forward; Y = C/T, R = A/G), 5′-AACRCGCTTAACAAAGCACTC-3′ (Reverse; R = A/G) and 5′-TAGTTGTGATGCWATCATGACTAG-3′ (Probe in 5′-FAM/ZEN/3′-IBFQ format; W = A/T). The reaction setup and the thermal cycling conditions were performed as described previously^25^.

#### Mechanism of action(s)

To investigate whether the tested drugs with low IC_50_ and high selectivity indices can directly hit the viral particle “virucidal effect” and/or interfer with viral adsorption and/or viral replication during virus replication cycle, plaque infectivity reduction assay was performed as described previously^8^.

### Docking studies

The drugs were subjected to four molecular docking studies using MOE 2019.012 suite^26^ to virtually examine their antiviral effects against both SARS-CoV-2 and MERS-CoV spike (S) and main protease (M^pro^) proteins and to propose their mechanism of action. The co-crystallized inhibitors of M^pro^ proteins were used as reference standards in each corresponding docking process in order to compare the binding scores and interactions of the tested antidepressant drugs.

#### Preparation of the tested FDA-approved antidepressant drugs (1–8)

Each drug was sketched using the MOE builder, adjusted for partial charges, 3D protonated, subjected to energy minimization process, and stored as (.moe) extension as described before^27–29^. Then, they were collected in four different databases, two of them containing only the prepared drugs (1–8) for both SARS-CoV-2 and MERS-CoV S proteins docking processes, the third one containing the prepared drugs together with the co-crystallized inhibitor (N3, 9) (1–9) for SARS-CoV-2 M^pro^ docking, and the fourth one containing the prepared drugs together with the two co-crystallized inhibitors (K36 9 and B1S 10) (1–10) for MERS-CoV M^pro^ docking. Each database was saved as an MDB file with (.mdb) extension to be used in the corresponding docking process.

#### Preparation of the SARS-CoV-2 and MERS-CoV S and M^pro^ pockets

The Protein Data Bank was used to extract the 3D X-ray structures of the S and M^pro^ of SARS-CoV-2 (PDB codes: 6VW1^30^ and 6LU7^31^, respectively) and MERS-CoV (PDB codes: 5YY5^32^ and 5WKJ^33^, respectively). Furthermore, the downloaded protein structures were subjected to the detailed preparation steps described earlier^34–36^ and including correction, 3D hydrogen addition, and energy minimization to be ready for docking steps^37–39^.

#### Docking processes of SARS-CoV-2 and MERS-CoV S and M^pro^ pockets

The previously mentioned four databases were used for four different docking processes into the prepared SARS-CoV-2 and MERS-CoV S and M^pro^ pockets, respectively. For each docking process, the corresponding database was introduced in place of the ligand in a general docking process. The dummy atoms were applied to select the docking site as the largest pocket for the prepared SARS-CoV-2 and MERS-CoV protein in each case. The program specifications were adjusted as described before in details^40–42^ and the docking process was initiated for each case. The tested compounds that achieved the best binding scores and modes for each docking process were selected for further investigations^43–45^.

Two program validation processes were carried out before the docking steps by redocking the co-crystallized inhibitors at their corresponding M^pro^ binding pockets^46–48^. The valid performance was confirmed by obtaining low RMSD values (< 2) between the co-crystallized and redocked inhibitor molecules in each case^49–55^. Moreover, the superimposed docked structure with the crystal structure was presented for each validation process in the supplementary data (Fig. SI1).

## Results

### Cytotoxicity and antiviral activity of the tested antidepressants drugs

**Figure 2:**
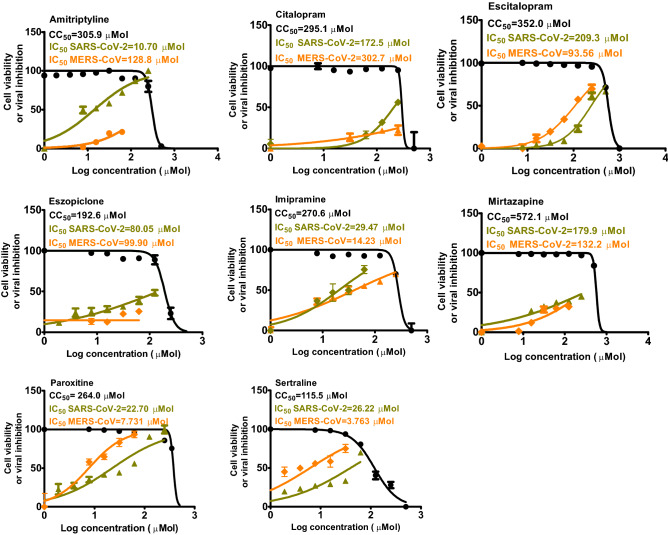
Determination of cytotoxic 50% (CC_50_) of selected antidepressants in Vero-E6 cells and inhibitory concentration 50% (IC_50_) of selected antidepressants against NRC-03-nhCoV in Vero-E6 cells and inhibitory concentration 50% (IC_50_) of selected antidepressants against NRCE-HKU270 in Vero-E6 cells. The CC_50_ and IC_50_ values were calculated using nonlinear regression analysis of GraphPad Prism software (version 5.01) by plotting log inhibitor versus normalized response (variable slope).

Half maximal cytotoxic concentration (CC_50_), inhibitory concentration 50 (IC_50_) and selectivity index (CC_50_/IC_50_) against SARS-CoV-2 and MERS-CoV were individually calculated (Fig. 2). The CC_50_ results for Citalopram, Escitalopram, Paroxetine, Sertraline, Mirtazapine, Amitriptyline, Imipramine, and Eszopicolone in Vero-E6 cells were 295 µM, 352 µM, 264 µM, 115.5 µM, 572 µM, 305.9 µM, 270 µM, and 192.6 µM respectively. The IC_50_ results of Citalopram, Escitalopram, Paroxetine, Sertraline, Mirtazapine, Amitriptyline, Imipramine, and Eszopicolone against SARS-CoV-2 were 172.5, 209.3, 22.7, 26.2, 179.9, 10.7, 29.4, 80 µM, respectively, while against MERS-CoV IC_50_ results were 302 µM, 93.5 µM, 7.7 µM, 3.7 µM, 132.2 µM, 128.8 µM, 14.23 µM, and 99.9 µM respectively (Fig. 2).

Awouafack et al*.* recommended an acceptance value of SI ≥ 10 for a selective bioactive compound^56^. Based on this criterion, we selected Amitriptyline and Paroxetine that showed SI 28.5 and 11.6 respectively, for SARS-CoV-2 for further bioassays. For MERS-CoV, Imipramine, Sertraline, and Paroxetine had the highest SI among tested compounds 19, 31.2, and 34.2 respectively, and were also selected for further in vitro studies.

### Time course analysis

**Figure 3:**
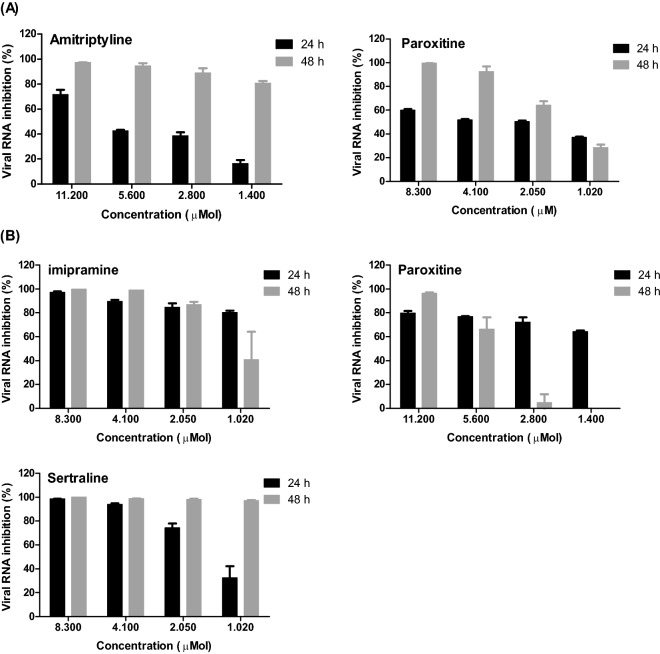
Impact of tested antidepressants on viral RNA copy numbers in infected Vero E6 cells (MOI 0.05) at 24 and 48 h post-treatment. Cells were infected with SARS-CoV-2 (**A**) and MERS-CoV (**B**) and then treated with various concentrations of the tested antidepressants.

Effects of antidepressant drugs (Amitriptyline and Paroxitine) on the propagation of SARS-CoV-2 after infection of Vero-E6 at 24 and 48 h were assessed by qRT-PCR. Remarkable differences were observed between viral inhibitory percentage at 24 h and 48 h post-treatment using tested antidepressant drugs. Amitriptyline and Paroxitine cause reduction in viral RNA copy number of SARS-CoV at MOI 0.05 (Fig. 3A).

The infected Vero-E6 cells with SARS-CoV-2 virus in the presence of different concentrations of Amitriptyline showed a reduction in viral RNA copy numbers assessed by qRT-PCR ranged from 80 to 97% in a dose-dependent fashion at 48 h post infection (78%). Also, presence of Paroxetine at concentration of 8.3 µM showed decreasing in viral inhibition (99%) compared to untreated and infected cells after 48 h post infection.

Effects of antidepressant drugs (Imipramine, Sertraline, and Paroxetine) on the propagation of MERS-CoV after infection of Vero-E6 were assessed by qRT-PCR at 24 and 48 h post infection. Imipramine, Sertraline, and Paroxetine cause reduction in viral RNA copy number of MERS-CoV virus at MOI 0.05 (Fig. 3B). Substantial differences were observed between viral inhibitory percentage at 24 h and 48 h post-treatment using tested antidepressant drugs.

### Mode of action

**Figure 4:**
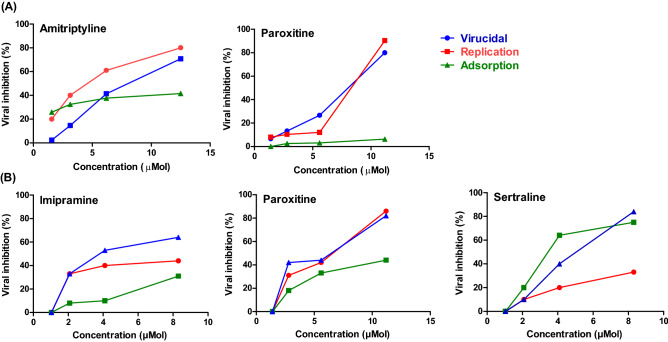
Mode of action for promising antidepressant drugs against SARS-CoV-2 (**A**) and MERS-CoV (**B**).

To determine the mechanism of action for the promising drugs towards tested viruses, it was necessary to examine the mode of action. Generally, candidate antiviral drugs can interfere with viral replication cycle by exerting direct virucidal effect or indirectly by blocking the viral adsorption into the host cell receptors or impairing intracellular viral replication^8^. Interestingly, Amitriptyline and Paroxitine had a combination of viral inhibitory effect on the tested SARS-CoV-2 with remarkable virucidal effect and effects on virus replication (Fig. 4A). Likewise, Imipramine and Paroxetine showed virucidal and replication potential combination mechanisms against MERS-CoV. On the other hand, Sertraline showed viral inhibitory effect against MERS-CoV at virucidal and adsorption mechanisms (Fig. 4B).

### Docking studies

By visualizing the M^pro^ binding pocket of SARS-CoV-2, it was found that it contains a co-crystallized inhibitor (N3), while that of MERS-CoV contains two co-crystallized inhibitors (K36 and K1S). All of them fitted inside the binding pocket of M^pro^ in an asymmetric manner. Molecular docking studies were performed for the eight examined drugs against both the S and M^pro^ pockets of SARS-CoV-2 and MERS-CoV in order to propose a suitable mechanism of action for their antiviral potentials.

The docking results on SARS-CoV-2 and MERS-CoV S and M^pro^ active sites showing their scores and binding modes were recorded in the four docking processes and compared to that of the docked inhibitors in case of M^pro^ docking in both viruses as depicted in Table 1.

**Table 1:** The binding scores and interactions of the examined FDA-approved antidepressant drugs (1–8) and the docked co-crystallized inhibitors (9) of M^pro^ pocket inside the S and M^pro^ binding sites of both SARS-CoV-2 and MERS-CoV.

No	Drug	R^a^	SARS-CoV-2		MERS-CoV	
			S^b^	Interactions	S^b^	Interactions
1	Amitriptyline	S	− 6.34	Thr347/pi-H Thr347/pi-H	− 6.35	Lys470/H-acceptor
		M^pro^	− 5.88	Tyr154/pi-H	− 6.15	–
2	Citalopram	S	− 6.44	His345/pi-pi	− 6.78	Ser426/H-acceptor Gln471/H-acceptor Lys470/pi-H
		M^pro^	− 6.63	Tyr154/pi-H	− 6.32	–
3	Escitalopram	S	− 6.51	–	− 6.62	Gln471/H-acceptor
		M^pro^	− 6.38	Tyr154/pi-pi	− 7.10	Met168/H-acceptor
4	Eszopiclone	S	− 6.87	Ala348/H-acceptor His345/H-acceptor His345/pi-pi	− 6.70	Gln471/pi-H Lys470/pi-H
		M^pro^	− 6.54	Arg298/H-acceptor Ser123/pi-H Tyr118/pi-pi	− 7.26	–
5	Imipramine	S	− 6.52	Ala348/H-acceptor His345/pi-pi	− 6.00	Gln471/H-acceptor
		M^pro^	− 6.00	–	− 6.29	Gly149/H-acceptor
6	Mirtazapine	S	− 5.65	Thr347/pi-H	− 6.44	–
		M^pro^	− 5.50	Tyr154/pi-H Tyr154/pi-pi	− 6.00	–
7	Paroxetine	S	− 6.62	–	− 6.79	Ser457/H-donor
		M^pro^	− 6.03	–	− 6.99	His166/pi-H
8	Sertraline	S	− 6.01	His345/pi-pi	− 6.17	Gln471/pi-H
		M^pro^	− 5.57	Tyr154/pi-H	− 5.99	–
9	Co-crystallized inhibitor	M^pro^	− 8.47	Gln192/H-donor Asn142/H-acceptor Met49/H-donor	− 7.58	Glu169/H-acceptor His166/pi-H
					− 7.89	His166/pi-H Phe143/H-pi

S, the spike protein; M^pro^, the main protease receptor.

^a^The receptor pocket.

^b^The score of a ligand inside the binding pocket (Kcal/mol).

Observing the docking scores and interactions of the eight examined drugs indicates their expected binding affinities and therefore the intrinsic activities as well. We decided to further examine the most promising four antidepressant drugs in their biological activities (Amitriptyline, Imipramine, Paroxetine, and Sertraline as depicted in Tables 1, 2, and 3. Moreover, the 3D pictures of the pocket interactions and positioning for other studied antidepressant drugs inside the S and Mpro pockets of SARS-CoV-2 and MES-CoV were represented in the supplementary information file (Tables SI 1 and SI 2, respectively).

**Table 2:**
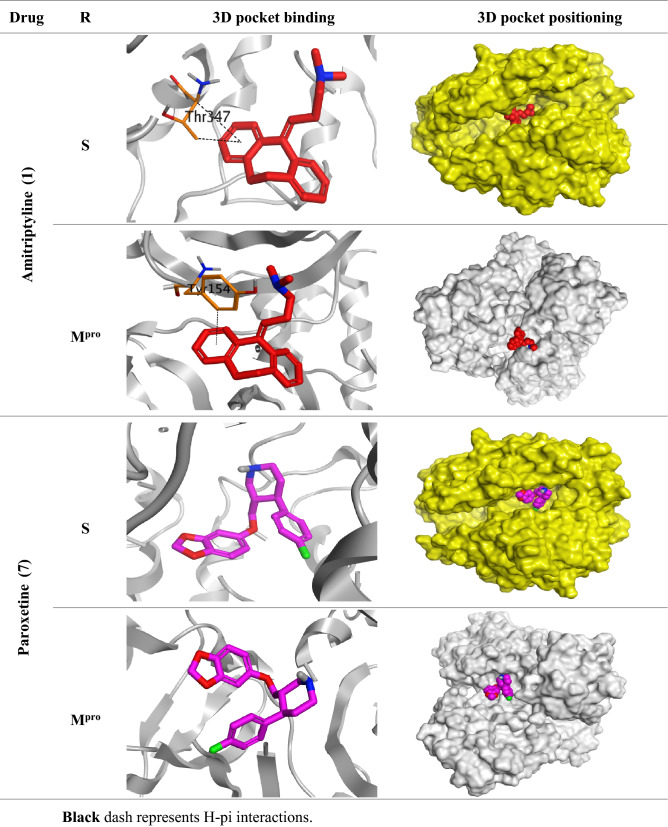
3D pictures of the pocket interactions and positioning for the most promising antidepressant drugs (Amitriptyline 1, and Paroxetine 7) inside the S and M^pro^ pockets of SARS-CoV-2.

Black dash represents H-pi interactions.

**Table 3:**
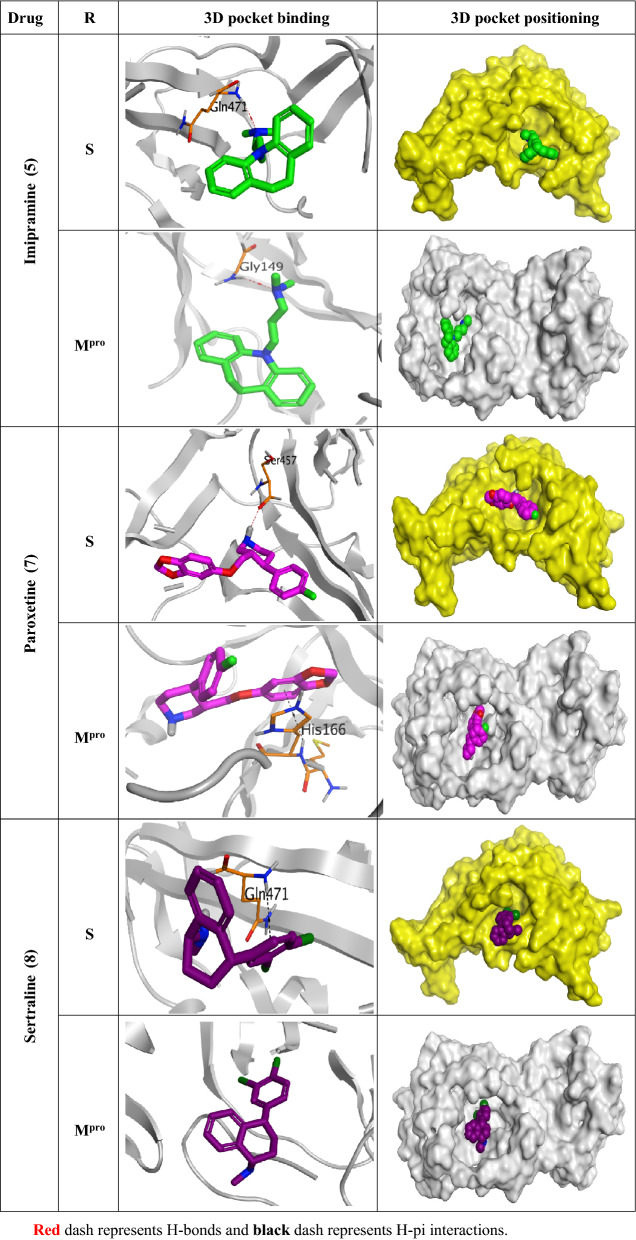
3D pictures of the receptor interactions and positioning for the most promising antidepressant drugs (Imipramine 5, Paroxetine 7, and Sertraline 8) inside the S and M^pro^ pockets of MERS-CoV.

Red dash represents H-bonds and black dash represents H-pi interactions.

Amitriptyline 1 achieved binding scores on the S and M^pro^ active sites of SARS-CoV-2 and MERS-CoV of − 6.34 and − 5.88 kcal/mol, respectively. It also formed two pi-H interactions with Thr347 amino acid inside the S pocket and one pi-H interaction with Tyr154 amino acid inside the M^pro^ pocket of SARS-CoV-2.

On the other hand, Imipramine binding scores were found to be − 6.00 and − 6.29 kcal/mol on the S and M^pro^ pockets of MERS-CoV, respectively. It bound Gln471 amino acid with a H-bond inside the S pocket, and also bound Gly149 amino acid with a H-bond inside the M^pro^ pocket of MERS-CoV.

Paroxetine 7 showed promising binding scores of − 6.62, − 6.03, − 6.79, − 6.99 kcal/mol on the S and M^pro^ active sites of SARS-CoV-2 and MERS-CoV, respectively. It got stabilized inside both the S and M^pro^ binding pockets of SARS-CoV-2 without any amino acid interactions indicating highly recommended affinity and stability as well. However, it formed one H-bond with Ser457 amino acid inside the S pocket, and one pi-H interaction with His166 amino acid inside the M^pro^ pocket of MERS-CoV.

Finally, Sertraline gave binding scores of − 6.17 and − 5.99 kcal/mol on the S and M^pro^ pockets of MERS-CoV, respectively. It formed one pi-H interaction with Gln471 amino acid inside the S pocket and showed no binding interactions inside the M^pro^ pocket of MERS-CoV.

Notably, the docked co-crystallized inhibitor of SARS-CoV-2 M^pro^ pocket (N3, 9) achieved a binding score of − 8.47 kcal/mol with the formation of three H-bonds with Gln192, Asn142, and Met49 amino acids, respectively. However, the two docked co-crystallized inhibitors of MERS-CoV Mpro pocket (K36, 9 and K1S, 10) got binding scores of − 7.58 and − 7.89 kcal/mol, respectively. The docked K36, 9 formed one H-bond with Glu169 and one pi-H interaction with His166 amino acid while the docked K1S, 10 formed one pi-H interaction with His166 and one H-pi interaction with phe143 amino acid.

## Discussion

Several clinical trials are currently conducted on COVID-19 patients to explore various drugs which may target different features of the disease pathophysiology^57^. One of the most important features of this disease is the excessive inflammation that occurs post infection with SARS-CoV-2. The excessive release of cytokines which is known as “cytokine storm” represents a serious condition that is responsible for respiratory dysfunction, pulmonary fibrosis, and eventually organ failure^58^. In a consistent manner, depressive episodes are commonly reported to be associated with elevated central and peripheral blood levels of pro-inflammatory cytokines and receptors including TNF-α, IL-1β, IL-6, C-reactive protein (CRP), sTNFR1, sTNFR2, soluble IL-2 receptors (sIL-2R)^59,60^. It is recently recognized that COVID-19 is associated with neurological and cerebrovascular disorders. Moreover, approximately 50% of the patients experience post recovery psychiatric diseases^61^, in which the diseases’ mechanisms are still unclear. However, latest reports suggested strong relationship between the inflammation involving the high cytokines levels and the psychiatric depressive disorders^62,63^. Consequently, the use of antidepressant drugs among the medications prescribed for COVID-19 patients is increasingly expected.

This observation led to explore any possible antiviral activity of a bundle of commercially available antidepressants in the market. The list of tested antidepressentants were also selected to cover variable popularity indices (PI) in real clinical practice including amitriptyline (PI = 1.06), paroxetine (2.09), citalopram (PI = 1.47), escitalopram (PI = 0.83) and sertraline (1.66)^64^.We envisioned that if a certain antidepressant drug has a potential inhibitory effect on SARS-CoV-2, this will be a double weapon for conquering COVID-19 not only by relieving of the associated psychiatric disorders but also through inhibition of virus replication. Antidepressant drugs, particularly some SSRIs and TCAs exhibit anti-inflammatory activities and they can easily diffuse in the central nervous system^65^. A recent report suggested that many antidepressant drugs may potentially reduce the activity of acid that subsequently may hinder SARS-CoV-2 infection to the epithelial cells^66^. Additionally, a clinical study has been performed on hospitalized COVID-19 patients and the authors signified the relationship between the intake of antidepressants and decreased mortality risks. Carpinteiro et al., reported that amitriptyline, imipramine, fluoxetine, sertraline, escitalopram, or maprotiline’s inhibition of acid sphingomyelin as well as genetic downregulation of it can prevent SARS-CoV-2 infection of either the cultured cells or the freshly separated human nasal epithelial cells^66^. In another clinical trial, treatment with SSRI fluvoxamine drug among high-risk outpatients with early diagnosed COVID-19 reduced significantly the hospitalization rate^67^. In line with previous studies, our in vitro and in silico studies demonstrated the potential ability of Amitriptyline and Paroxetine to effectively inhibit SARS-CoV-2. Also, Imipramine, Sertraline, and Paroxetine showed antiviral activity against MERS-CoV. The aforementioned docking discussions of the FDA-approved antidepressant drugs (amitriptyline, imipramine, paroxetine, and sertraline) against both SARS-CoV-2 and MERS-CoV pockets (S and M^pro^) clarified greatly the binding affinities and therefore the expected intrinsic activities of the previously mentioned drugs towards the SARS-CoV-2 and MERS-CoV. Taking into consideration all this, besides the high safety records proved by the post marketing experience of antidepressant drugs, we encourage clinical trials for repositioning the use of these antidepressants to manage COVID-19 disease.

## Conclusion

This study proposed the effective antidepressants drugs against SARS-CoV-2 (Amitriptyline and Paroxetine) and MERS-CoV (Imipramine, Sertraline, and Paroxetine), to be further evaluated by extra in vitro and in vivo experiments in order to obtain an effective antidepressant therapeutic targeting coronaviruses to be recommended to patients suffering from depression. Furthermore, these drugs could be used as lead compounds for further optimization to get more promising molecules with promising activities towards SARS-CoV-2.

One limitation of this study is that it did not cover all commercial antidepressants especially the novel unclassified antidepressants including ketamine, agomelatine and vortioxetine. Agomelatine is a novel antidepressant as well as a potent agonist of melatonin (MT), MT1 and MT2 receptor types and an antagonist of the serotonin (5HT)^68^. Vortioxetine is also a novel antidepressant capable of improving depressive and cognitive symptoms associated with major depressive disorder (MDD)^69^, while Ketamine is a rapid-acting and novel therapeutic treatment for treatment-resistant depression, which has also been demonstrated to attenuate symptoms of anhedonia^70^. Therefore we highly propose the testing of the in vitro antiviral activity of these novel antidepressants against COVID-19 in future studies.

## Supplementary Information

Supplementary Information.

## Data Availability

All data analysed during the current study are available from the corresponding author on reasonable request. Data that support the findings of this study are available within the paper and its supplementary data.
