# Supplementary material for: Robust antiviral activity of commonly prescribed antidepressants against emerging coronaviruses: in vitro and in silico drug repurposing studies
Source: Sci Rep. 2022 Jul 28;12:12920. doi: 10.1038/s41598-022-17082-6 (PMC9331004; doi:10.1038/s41598-022-17082-6)
Supplement: Supplementary file 1 — Supplementary Information. [file 41598_2022_17082_MOESM1_ESM.docx]

**Robust Antiviral Activity of Commonly Prescribed Antidepressants against Emerging Coronaviruses: *In Vitro* and *In Silico* Drug Repurposing Studies**

Omnia Kutkat ^1,†^, Yassmin Moatasim ^1,†^, Ahmed A. Al‐Karmalawy ^2^, Hamada S. Abulkhair ^2,3^, Mokhtar R. Gomaa ^1^, Ahmed N El-Taweel ^1^, Noura M. Abo Shama ^1^, Mohamed GabAllah ^1^, Dina B. Mahmoud ^4^, Ghazi Kayali ^5,6^, Mohamed A. Ali ^1^, Ahmed Kandeil ^1,*^ and Ahmed Mostafa ^1,*^

**Supplementary Information**

**Figure SI1.** 2D and 3D overlay diagrams revealing the superimposition of the native co-crystallized and the redocked inhibitors at MERS-CoV and SARS-CoV-2 M^pro^ binding pockets.

| **MERS-CoV** | |
| --- | --- |
| **2D** | **3D** |
| 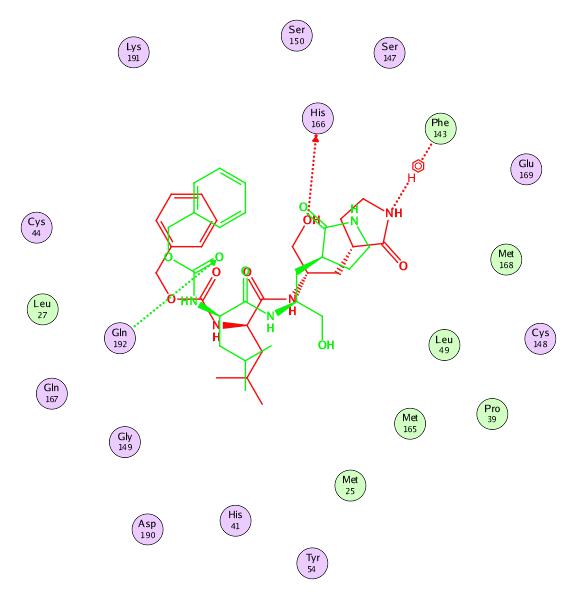 | 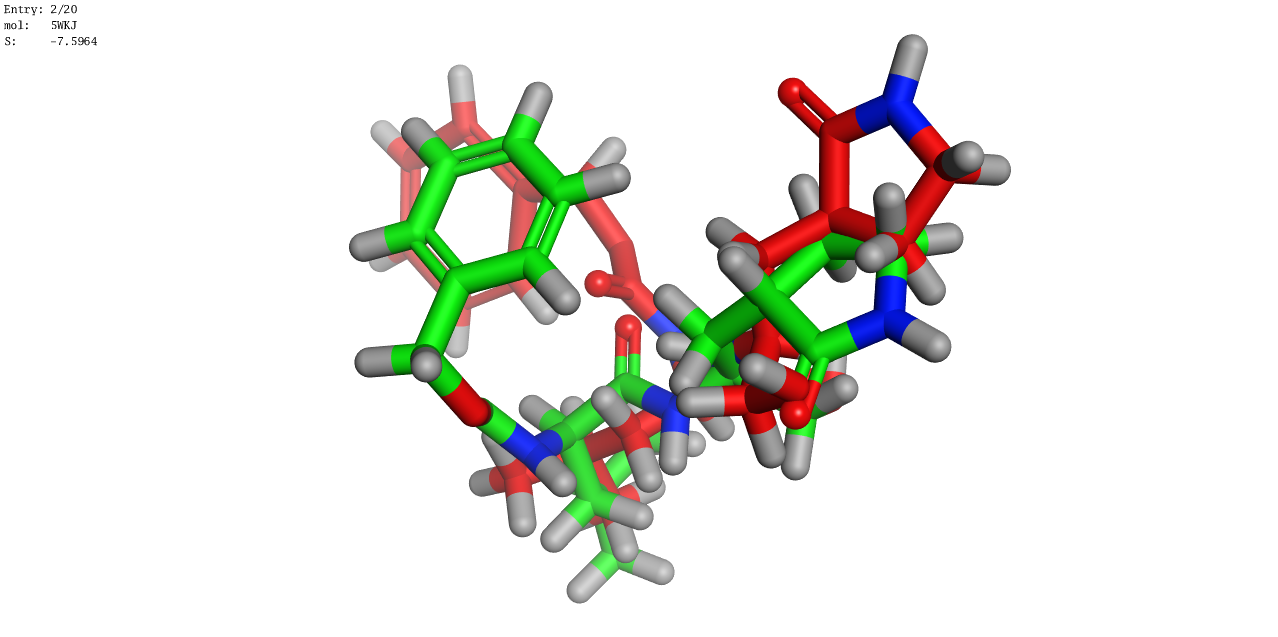 |
| **SARS-CoV-2** | |
| **2D** | **3D** |
| 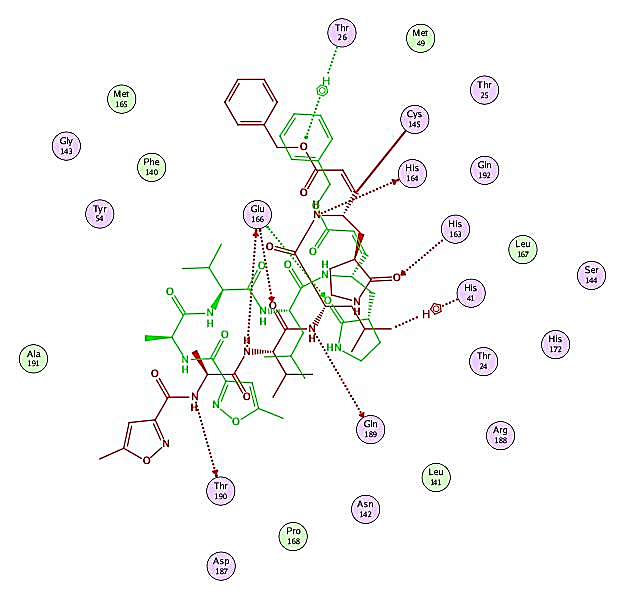 | 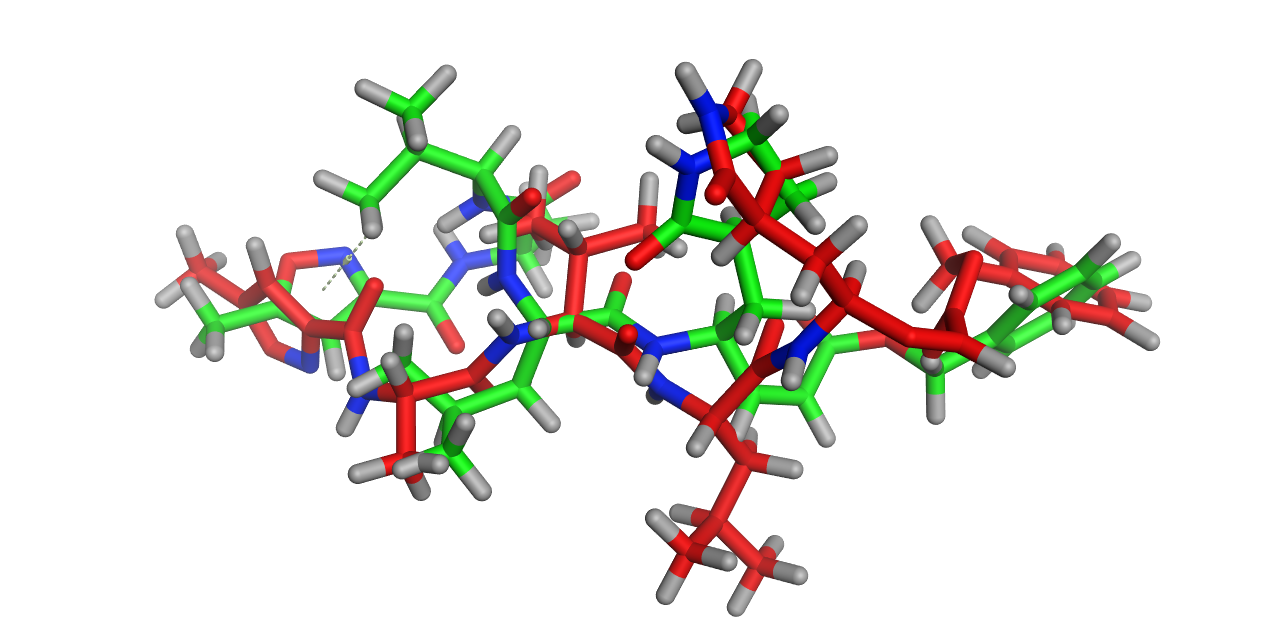 |

**Table SI 1.** 3D pictures of the pocket interactions and positioning for the antidepressant drugs (imipramine **5** and sertraline **8**) inside the S and M^pro^ pockets of SARS-CoV-2.

| **Drug** | **R** | **3D pocket binding** | **3D pocket positioning** |
| --- | --- | --- | --- |
| Imipramine  (**5**) | S | 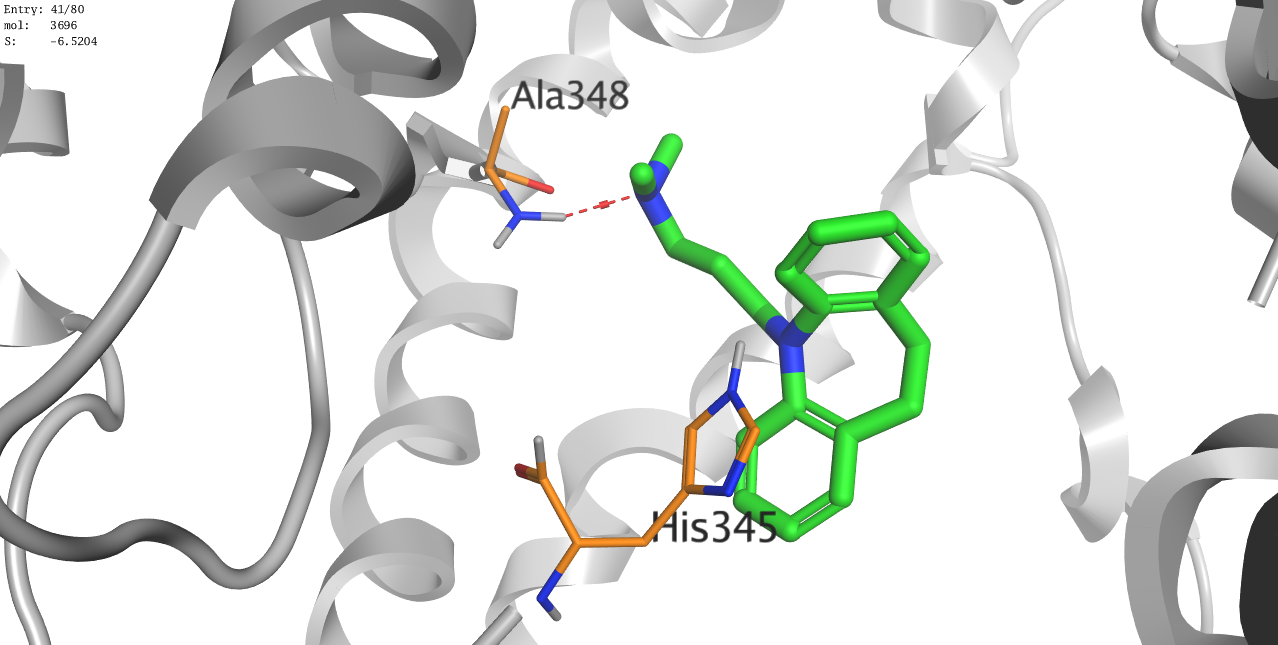 | 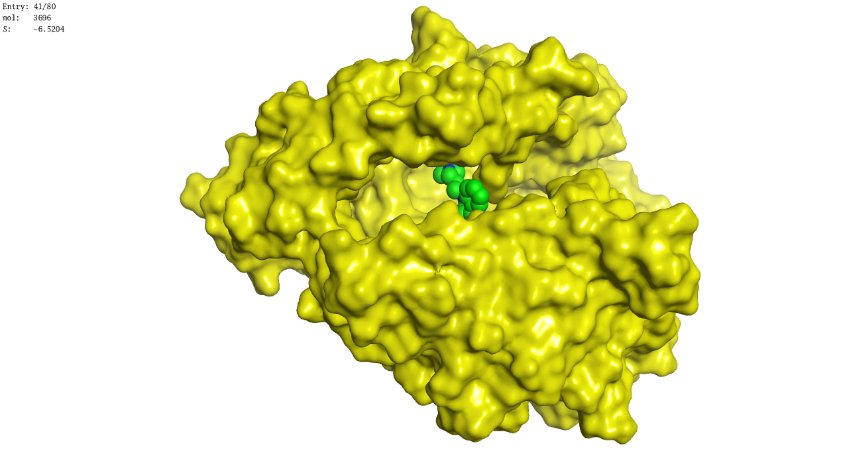 |
|  | M^pro^ | 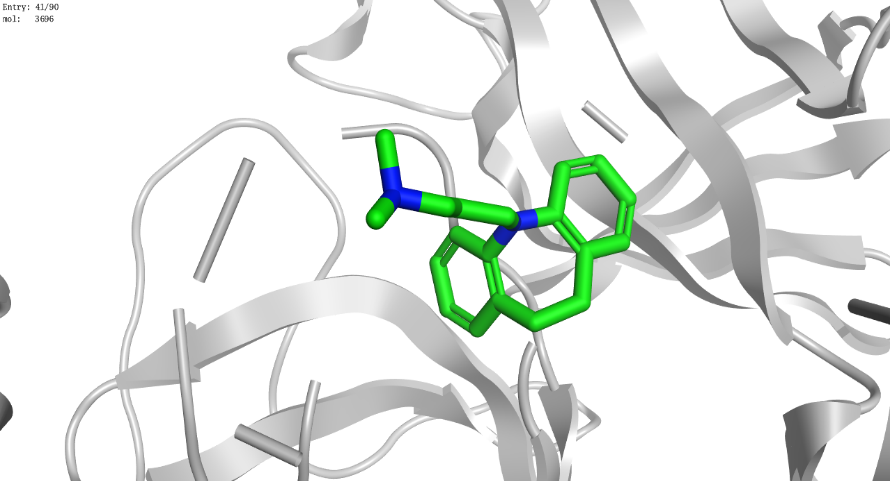 | 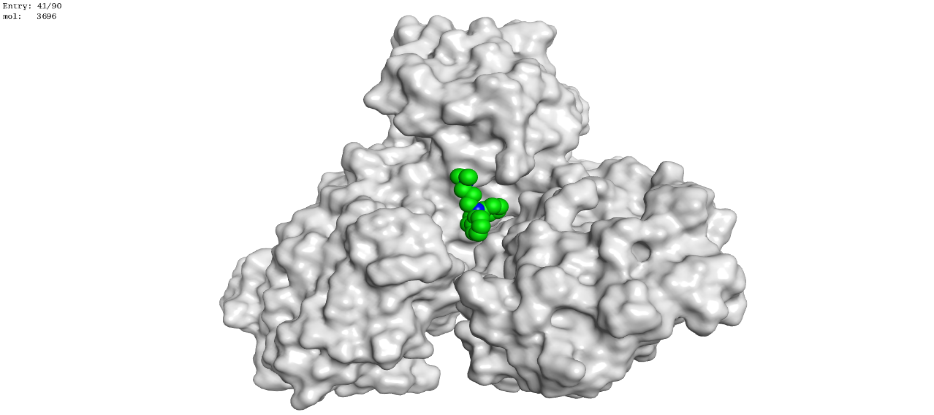 |
| Sertraline  (**8**) | S | 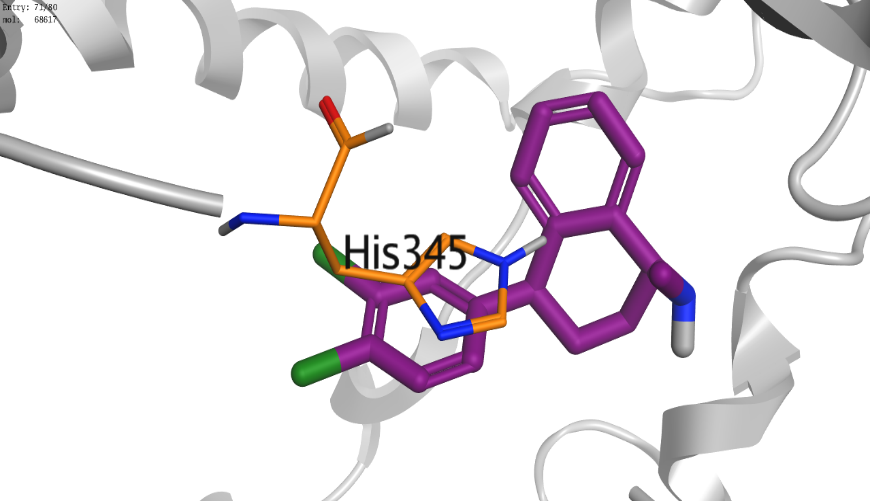 | 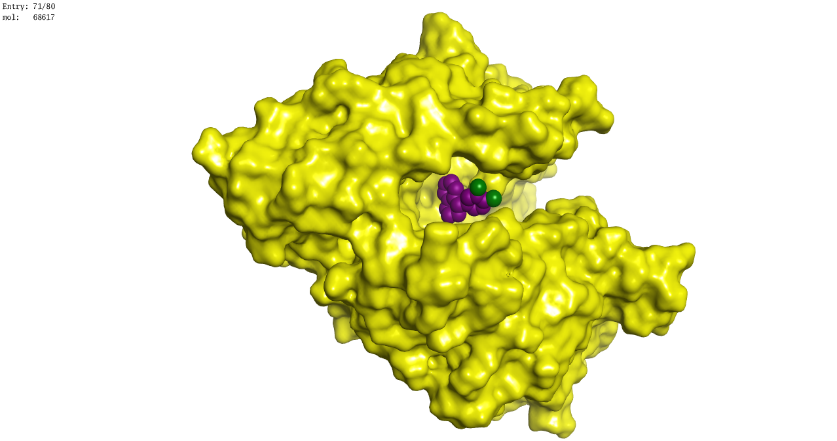 |
|  | M^pro^ | 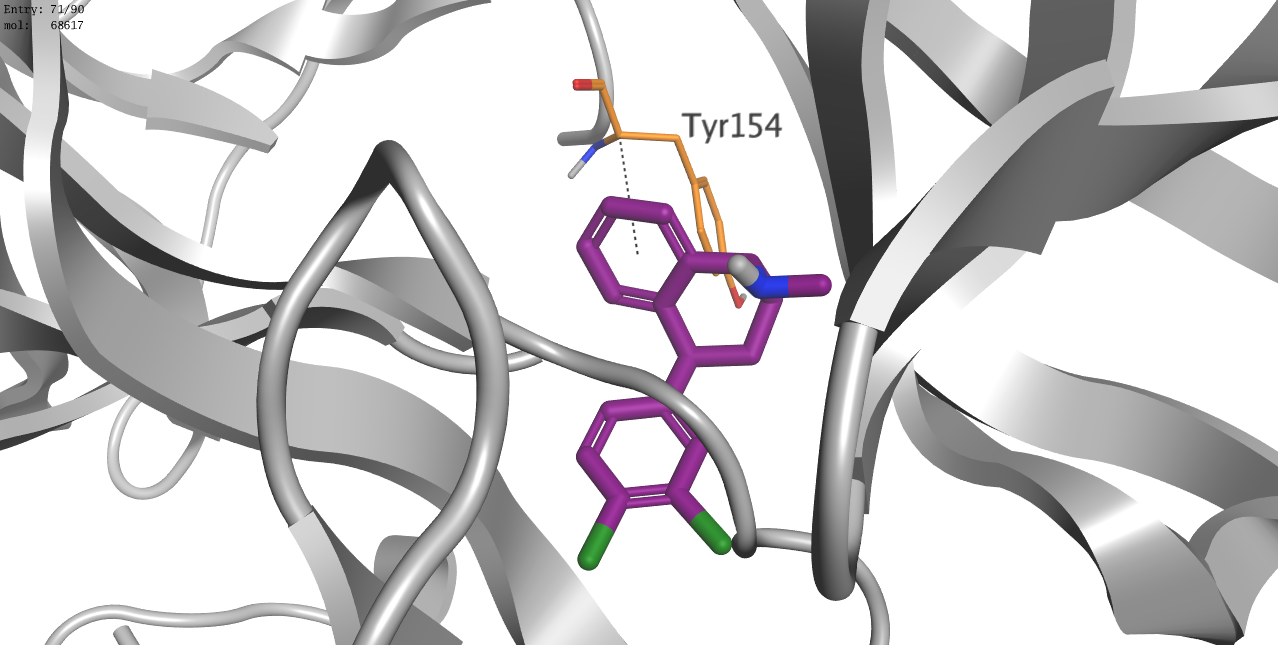 | 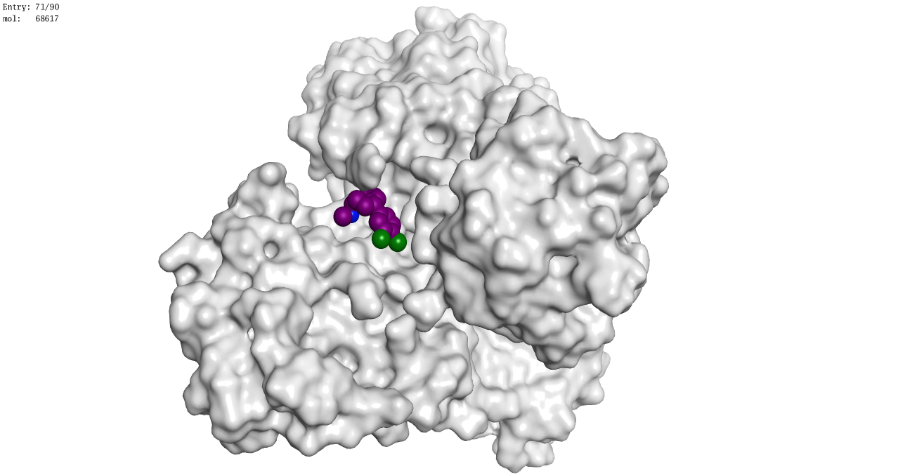 |

**Red** dash represents H-bonds and **black** dash represents H-pi interactions.

**Table SI 2.** 3D pictures of the pocket interactions and positioning for the antidepressant drug (amitriptyline **1**) inside the S and M^pro^ pockets of MERS-CoV.

| **Drug** | **R** | **3D pocket binding** | **3D pocket positioning** |
| --- | --- | --- | --- |
| Amitriptyline  (**1**) | S | 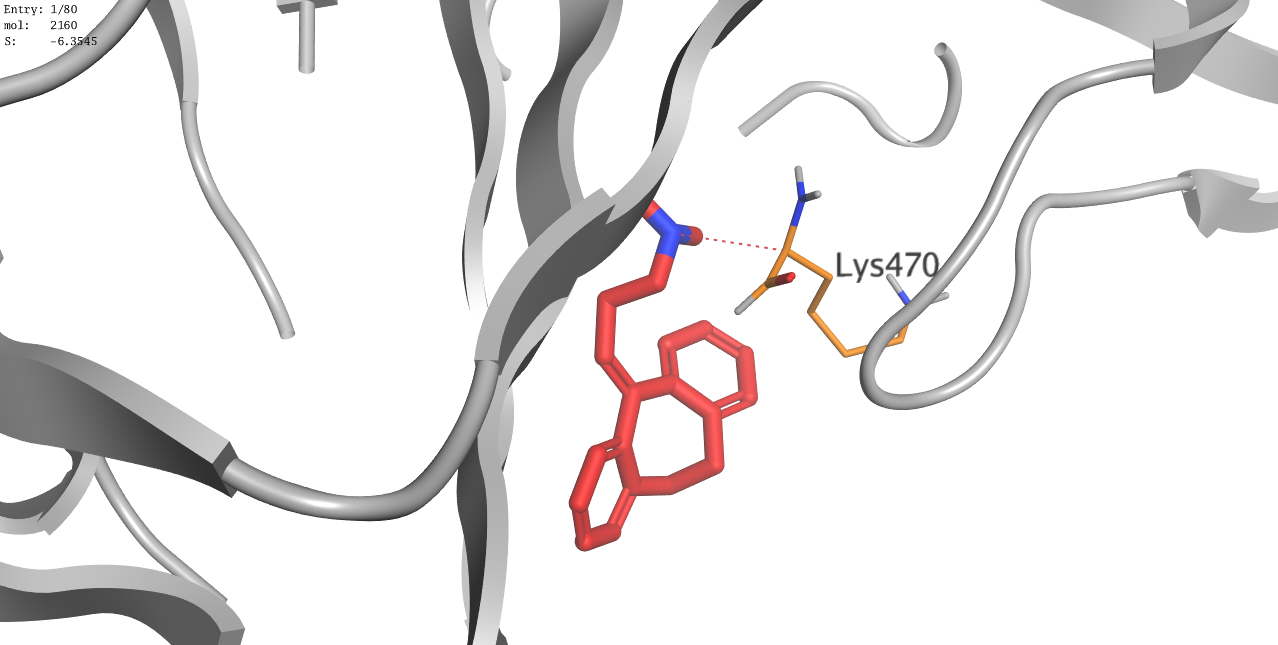 | 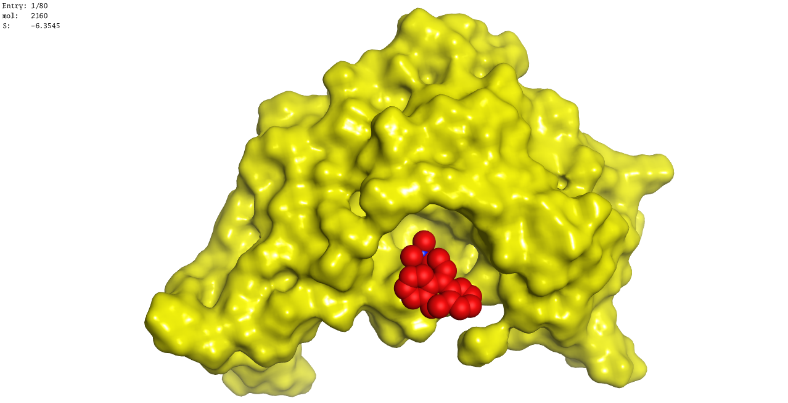 |
|  | M^pro^ | 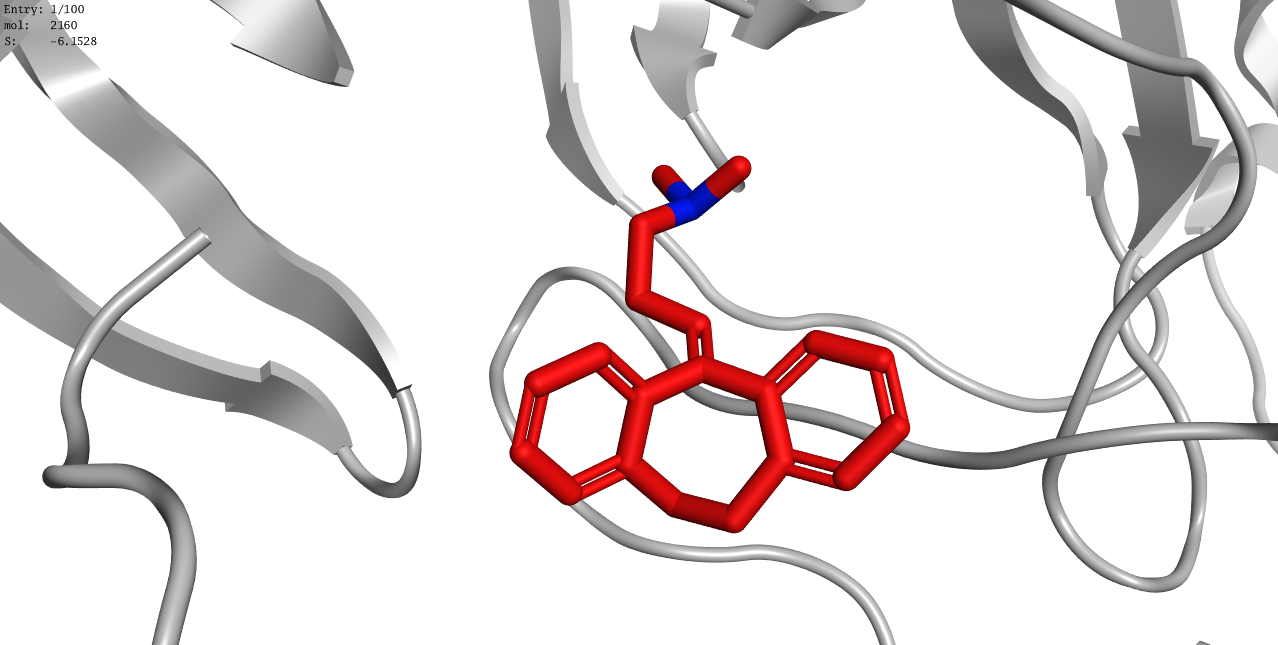 | 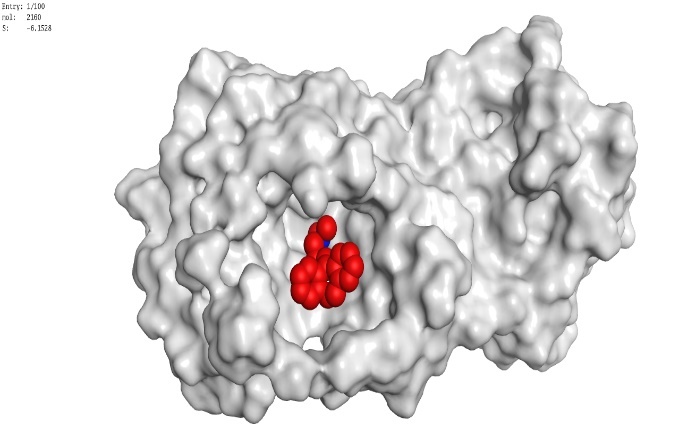 |

**Red** dash represents H-bonds and **black** dash represents H-pi interactions.
